# Ionizing radiation and bone quality: time-dependent effects

**DOI:** 10.1186/s13014-019-1219-y

**Published:** 2019-01-22

**Authors:** Pedro Henrique Justino Oliveira Limirio, Priscilla Barbosa Ferreira Soares, Eduardo Tadashi Pinto Emi, Camila de Carvalho Almança Lopes, Flaviana Soares Rocha, Jonas Dantas Batista, Gustavo Davi Rabelo, Paula Dechichi

**Affiliations:** 1 Integrated Dental Clinic Program, Faculty of Dentistry, Federal University of Uberlândia, Avenida Pará s/n°, Campus Umuarama, Bloco 4L, Bairro Umuarama, Uberlândia, Minas Gerais 38.400-902 Brazil; 2 Faculty of Dentistry, Federal University of Juiz de Fora, Faculdade de Odontologia – Campus Universitário - Bairro Martelos –, Juiz de Fora, Minas Gerais 36036-300 Brazil

**Keywords:** Cortical bone, Radiation, ionizing, Biomechanics, Bone matrix

## Abstract

**Background:**

The aim of this study was to evaluate the ionizing radiation (IR) effects on rat bone 30 and 60 days after irradiation.

**Methods:**

Wistar rats were submitted to IR (30 Gy) on the left leg and were euthanized after 30 and 60 days. The legs were divided into four groups according to the treatment and euthanization time: C30 and C60 (right leg–without IR), IR30 and IR60 (left leg-with IR).

**Results:**

CT analysis showed more radiodensity in C60 compared with other groups, and IR60 showed more radiodensity than IR30. In histomorphometric analysis, C30 showed lower bone matrix values compared with IR30 and C60. Lacunarity analyses showed more homogeneous bone channel distribution in C30 than IR30. ATR-FTIR showed decrease in ratio of mature and immature crosslinks in IR30 compared with C30. Crystallinity Index was decrease in IR60 compared with C60. The Amide III + Collagen/HA ratio was increased in C60 compared with C30; however this ratio decreased in IR60 compared with IR30. Biomechanical analysis showed lower values in IR groups in both time.

**Conclusions:**

IR damaged bone quality and decreased stiffness. Moreover, the results suggested that the deleterious effects of IR increased in the late time points.

## Introduction

Radiotherapy has been proven to be successful treatment for local and regional neoplastic lesions, but it may adversely affect normal tissues [1]. The high vulnerability to ionizing radiation (IR) has previously been documented in some bones (pelvis, sternum, vertebra, clavicle, femoral head, and mandible) [2]; leading to deleterious effect on the bone metabolism and healing, increasing the risk for infection, atrophy, pathological fractures, and osteoradionecrosis [1]. However, the deleterious effects of IR on healthy bone continue to be a cause for concern.

The complications in irradiated bone are dose dependent [3], and directly affect cell activity and repopulation capacity. Bone cells proliferate slowly, thus they are less affected by small fraction radiation or low total dose rates, being more susceptible to injury with increased doses [4]. Radiation injuries in normal tissue are commonly referred to as complications in different times. Late effects are typically reported after a latent period, and may occasionally develop years after exposure to radiation [5, 6].

Studies have shown that IR applied in treatment of primary and secondary bone malignancy leads to hypocellularity, alterations of the Haversian systems and bone matrices [7, 8]. These changes result in deteriorated bone formation, with decreased osteoblast proliferation and differentiation [9], induction to cell-cycle arrest and direct cell death [1], damage of microvascular structures [10] and decreased collagen production [11].

Bone is a multiphase hierarchical structure composed of organic and mineral components, and water [12]. Some studies have shown that collagen molecules denature due to water radiolysis, which produces free radicals, affecting the collagen interfacial bond with hydroxyapatite (HA) [13]. The microarchitecture and mechanical properties of bone are dependent on the specific arrangement and interaction between the organic matrix and mineral apatite crystals that form a carefully designed composite material [14].

Alterations in both intrinsic (mineral and collagen quality) and extrinsic (microarchitecture, bone mass and bone mineral density) determinants of bone strength will influence the mechanisms of repair and resistance [15, 16]. All these deleterious effects caused by IR will have an influence on bone mechanical properties, since they are essential to maintain the overall mechanical competence of bone [15, 16]. However, the substantial contribution of collagen network and mineral crystal structure to the structural and mechanical alterations in bone induced by IR are not fully understood.

To understand how bone tissue is damaged by radiation, it is necessary to use several types of analysis, considering changes in bone microarchitecture, composition of matrices, and mechanical properties. The aim of the present study was to evaluate the effects of IR on bone matrices, biomechanical properties, radiodensity, collagen and crystalline HA content in the femur and tibia of rats at 30 and 60 days after exposure to radiation.

## Material and methods

Ten healthy male Wistar rats (*Rattus norvegicus*), weighing between 250 and 300 g (10 weeks of age), were included in the study. This study was approved by The Institutional Science and Ethics Committee on the use of animals (Protocol 022/12), and was conducted in accordance with the provisions of Law No. 11,794, Decree No. 6.899 and complementary legislation of the Brazilian National Council for the Control of Animal Experimentation (CONCEA) guidelines. The animals were kept in cages, in a 12 h:12 h light-dark cycle, and controlled temperature conditions (22 ± 2 °C), with standard food and water ad libitum. All animals were submitted to IR on the left leg. The tibiae and femur were removed, and according to the treatment and euthanization time, the specimens were separated into four groups (*n* = 5): control 30 days (C30), irradiated 30 days (RX30), control 60 days (C60) and irradiated 60 days (RX60).

Before irradiation, the animals were anaesthetized by an intraperitoneal injection of 100 mg/kg ketamine 10% and 7 mg/kg xylazine 2% hydrochloride. The left leg was positioned laterally and fixed using a wooden stick and adhesive tape. A 1.5 cm thick wax bolus was positioned on the leg. Both the left leg femur and tibia were irradiated in a single anterior field (RX30 and RX60 groups). The right legs did not receive radiation and were designated to be the control group (C30 and C60 groups). The beam was collimated and irradiation was delivered using a linear accelerator (Varian Clinac® 600C S/N 0310, Palo Alto, CA, USA) with a total dose of 30 Gy in one session [17]. The animals were euthanized 30 or 60 days after radiation. The tibiae and femurs were removed by disarticulation, immediately placed in gauze with physiological saline solution and kept frozen in a freezer (− 20 °C). Twenty-four hours before the analyses, the tibiae and femur were defrosted and placed in phosphate buffered saline. The total tibiae were scanned by computed tomography (CT) and segmented in the mid-diaphysis. The distal diaphyses were decalcified in 10% EDTA and embedded in paraffin; and the proximal diaphysis was used for the Attenuated Total Reflectance Fourier Transform Infrared Spectroscopy (ATR-FTIR) analysis. The femurs were used in biomechanical analyses.

### Computed tomographic analysis

**Fig. 1:**
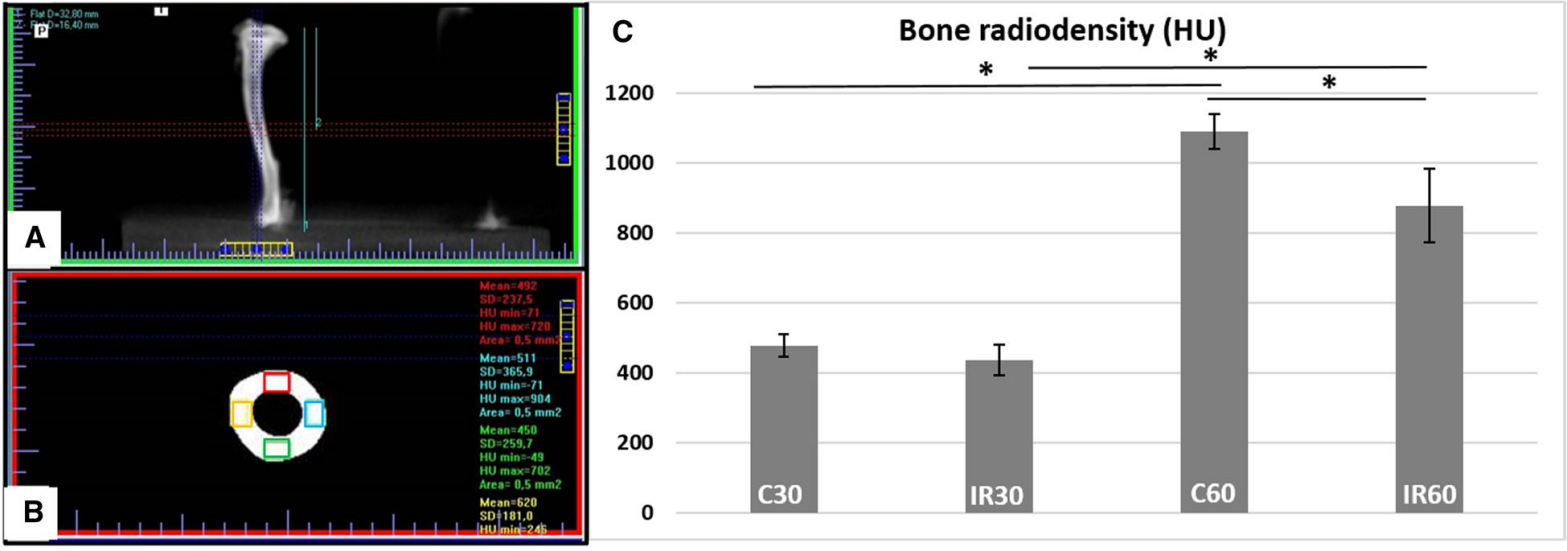
Computed Tomographic analysis. **a** Measure of the tibia length and determination of its median region, **b** Transverse section in middle of tibia and demarcation of the regions of interest in the cortical bone (colored rectangles). **c** Radiodensity analysis results. (*p < 0.05)

The total tibiae were positioned perpendicular to the basal surface and scanned with a Cone-Beam 3D scanner (Gendex®, GX-CB500-ICAT) at 7 mA, 120kvp and 0.125 mm voxel resolution (Fig. 1a). In the image of the transverse section, four rectangular marks measuring 0.5 mm^2^ were delineated in the middle of each tibia, representing the regions of interest (ROI) in the cortical bone (Fig. 1a, b). Bone radiodensity of ROIs was calculated using calibration by the Hounsfield scale, obtained by using specific software (i-CAT® Vision, Imaging Sciences International, Penn Road, Hatfield, PA, USA.).

### Histological and Lacunarity analysis

The distal mid-diaphyses (decalcified and embedded in paraffin) were sectioned into 5 μm-thick transverse sections that were stained with hematoxylin and eosin (H&E) for qualitative and quantitative bone matrix evaluations. Of each section, seven images captured of each of Groups C30 and IR30, and ten images of C60 and IR60 groups were digitized, for analysis of the entire cortical. For quantitative analyses, screen shots of the histological images were merged, and the blood vessels were erased using Photoshop CS6 software (Adobe®, Adobe System Inc., San Jose, CA/USA). Then, the images were converted into binary images by means of HL Image 2005^++^ software (Western Vision®, Salt Lake City, UT, USA) and the percentage of bone matrix was analyzed using HL Image 2005^++^ software. Lacunarity was calculated by the mathematical quantitative method that evaluates bone channel network features, considering the presence, size and spatial distribution of the channels within the bone matrix [18].

### ATR – FTIR and biomechanics analyses

The proximal mid-diaphysis was sectioned with a diamond disk under constant irrigation to obtain three fragments measuring 2 × 2 mm; resulting in 15 fragments per group. The bone composition was analyzed using ATR-FTIR, Vertex 70 (Bruker, Ettlingen, Germany) equipped with an accessory that allowed spectrum acquisitions in the Attenuated Reflectance (ATR) mode. The sample was scanned 32 times, and the spectrum acquired was the average of all scans. Three spectra were obtained from each tibia. The bone fragment was placed against the diamond crystal of the ATR-FTIR unit and pressed with a force gauge at a constant pressure to facilitate contact. The spectra were recorded in the range of 400–4000 cm^− 1^ at a 4 cm^− 1^ resolution. Data were recorded and analyzed with OPUS 6.5 software (Bruker, Ettlingen, Germany). After recording the spectra, vector normalization and baseline correction were performed.

The ATR-FTIR spectra were further analyzed by calculating the following parameters: Amide I band (AI) (Collagen ratio between the mature pyridinoline crosslink peaks (PYR) – 1660 cm^− 1^ and immature crosslinking dihydroxynorleucina (DHLNL) - 1690 cm^− 1^); Crystallinity Index (CI) (The intensity ratio of peaks 551 and 597 cm^− 1^ for 588 cm^− 1^); Matrix-to-mineral ratio: Amide I + II/Hydroxyapatite (HA) (M:MI) (The ratio between integrated areas of amide I + II (1520–1720 cm^− 1^) for HA (916–1180 cm^− 1^)) and Amide III + Collagen/HA (M:MIII) (The ratio between integrated areas of amide III (1210–1270 cm^− 1^) with two collagen bands (1269–1296 cm^− 1^ and 1180–1213 cm^− 1^) for HA (916–1180 cm^− 1^), in accordance with previous studies [19].

The femurs were first analyzed by means of a three-point bending test until failure, using universal-testing machine (EMIC DL 2000, EMIC Equipamentos e Sistemas de Ensaio Ltda, Sao José dos Pinhais, Brazil). The femur was placed horizontally on the two holding fixtures (16 mm) in the machine; the upper device load was applied in the middle of the diaphysis at a loading rate of 1.0 mm/min. The load and displacement data were recorded, subsequently, load vs. displacement curves were plotted. The results were finally calculated as the flexural modulus (FM) (GPa) and flexural strength (FS) (MPa) values. Femurs fractured after the mechanical tests were maintained in phosphate buffered saline until the Indentation analysis [19].

In the Indentation test, a fragment of 2 mm was removed from the fracture area (created in the three-point bending test), using a diamond disk under constant irrigation. The distal and proximal femur fragments were embedded in polyester resin (Instrumental Instrumentos de Medição Ltda, São Paulo, SP, Brazil) using a metal device (Metalon; Metalon Pooled Industries, Nova Iguaçu, RJ, Brazil) measuring 50 mm long, 30 mm wide and 10 mm high. The diaphyses were positioned perpendicular to the basal surface. After being embedded in polyester resin, the surfaces were finished using 600, 800, 1200 and 2000 grit silicon-carbide papers (Norton, Campinas, SP, Brazil) and polished with metallographic diamond pastes (6, 3, 1, ¼ μm, Arotec, São Paulo, SP, Brazil). The metallic device with diaphysis included were washed between polished papers in an ultrasound bath (Cristofoli, Campo Mourão, PR, Brazil) with absolute alcohol for 10 min to remove the debris (Soares, 2014). The Vickers Hardness (VHN) (MPa) and Elastic Modulus (GPa) of the bone were assessed by using a Microhardness dynamic identer (CSM Micro-Hardness Tester; CSM Instruments, Peseux, Switzerland). The indentation was made with controlled force, whereby the test load was increased or decreased at a constant speed ranging between 0 and 200 mN in 60-s intervals. The maximum force of 200 mN was held for 15 s. Five continuous indentations were made at a distance of 0.5 mm between each other perpendicularly to the cortical bone transverse ring interface [20].

Analysis was performed using statistical software Sigma Plot 13.1® (Systat Software Inc., San Jose, CA, USA). The results obtained were submitted to the Kolmogorov-Smirnov normality test and Two-Way Anova followed by the Tukey test. Differences were considered statistically significant when α < 0.05.

## Results

CT analyses showed that C60 (1090.8 ± 50.6) had an increased radiodensity value, compared with those of the other groups C30 (478.14 ± 31.27), IR30 (436.42 ± 43.22) and IR60 (877.9 ± 106) (*p* < 0.01). In addition, IR60 showed increased radiodensity compared with IR30 (*p* < 0.01) (Fig. 1c).

**Fig. 2:**
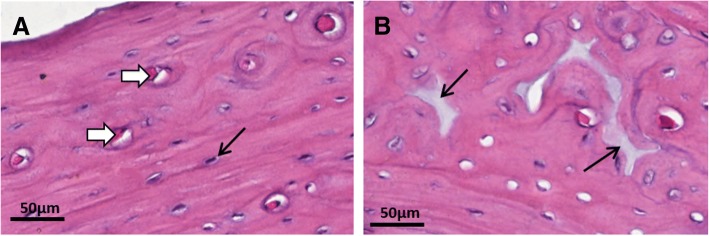
Histological images representative of the cortical bone. **a** Bone channels (white arrow) and osteocytes (black arrow) included in the bone matrix observed in the groups. **b** Amorphous areas with basophilic staining (arrow) observed in C60, IR30 and IR60 groups. Hematoxylin and Eosin stain

The histological analysis showed cortical bone with Haversian channels and osteocytes included in bone matrix in all groups. In some sections, it was possible to identify the cement line at the limit of the osteons, with the majority of them having only a few concentric lamellar layers. Channels containing blood vessels were noted, spatially distributed throughout the entire cortex (Fig. 2a). Basophilic tidemarks were observed in the groups IR30, C60 and IR60, accompanied by amorphous basophilic areas (Fig. 2b).

**Fig. 3:**
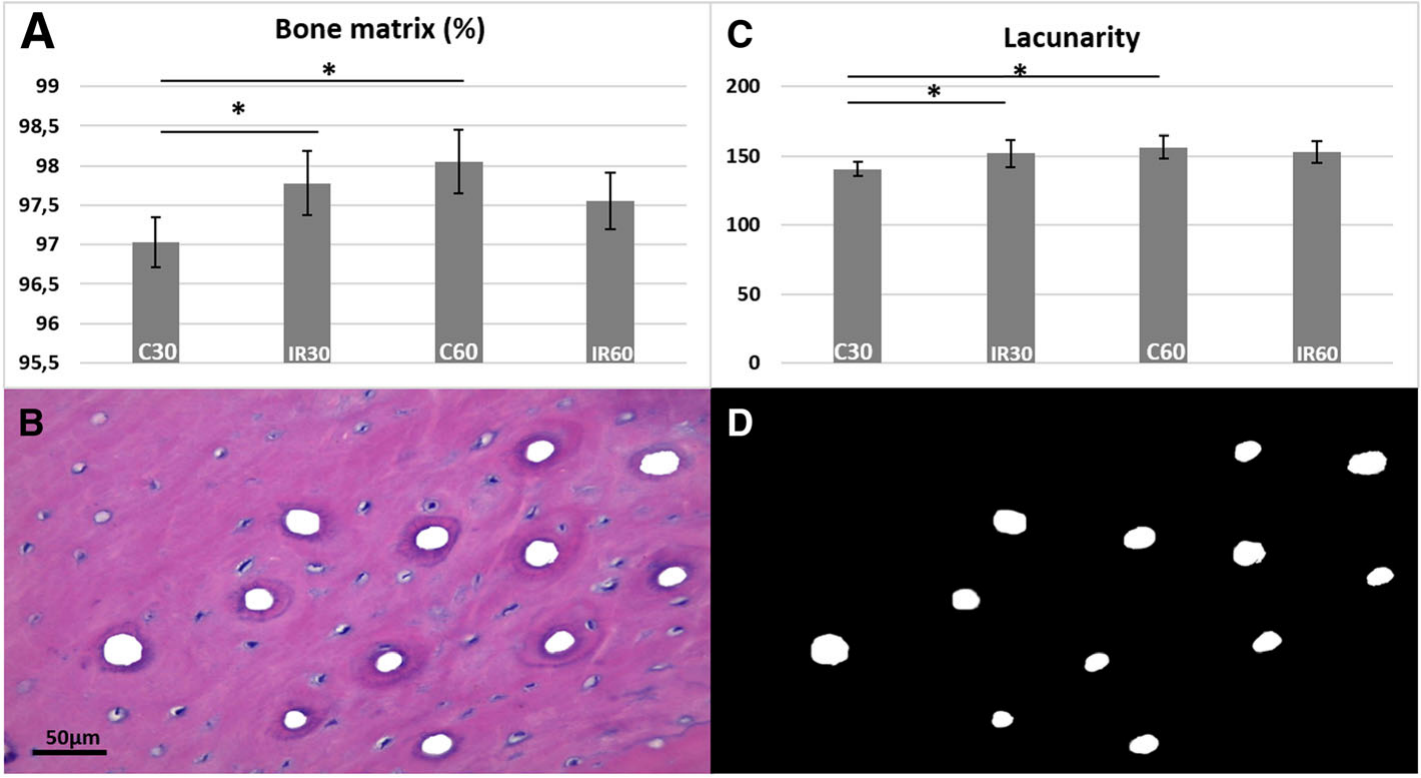
Histological and Lacunarity analysis. **a** Bone matrix percentage. **b** Histological image of cortical bone after delimitation and subtraction of the channels. **c** Lacunarity analysis. **d** Binary image obtained after bone channel segmentation. (*p < 0.05) HE stains

The histomorphometric analyses showed a lower percentage of bone matrix in C30 (97.03 ± 0.31) compared with IR30 (97.78 ± 0.40) and C60 (98.04 ± 0.3) (*p* < 0.01). However, there was no difference between C60 (98.04 ± 0.3) and IR60 (97.55 ± 0.3) (*p* > 0.05) (Fig. 3a, b). In the Lacunarity analysis, C30 (140.58 ± 5.04) showed more homogeneous distribution of channels than IR 30 (151.77 ± 9.78) and C60 (156.41 ± 8.02) (*p* < 0.04). There was no significant difference between C60 and IR60 (Fig. 3c, d).

The ATR-FTIR results are shown in Table 1. In the spectra, maintenance of the main bands characteristic of bone components were observed. ATR-FTIR showed a decreased amide I band ratio in IR30 compared with C30 (*p* = 0.04). In the time interval of 60 days, there was no significant difference in the collagen ratio (*p* = 0.37). Crystallinity Index (CI) was decreased in IR60 compared with C60 (*p* = 0.04); and there was no difference in 30 days (*p* = 0.18). In the analysis of the matrix-to-mineral ratio, the Amide I + II/HA showed no statistical difference between groups and time intervals (*p* > 0.09). The Amide III + Collagen/HA was increased in C60 compared with C30 (*p* < 0.01), however in IR60 it was decreased compared with IR30 (*p* < 0.01).

**Table 1:** The means and standard deviation values of ATR-FTIR analysis in the cortical tibiae

Tests	C30	IR30	C60	IR60
AI	3.06 ± 0.2^Ba^	2.52 ± 0.2^Aa^	3.03 ± 0.34^Ba^	2.69 ± 0.2^Aa^
CI	3.69 ± 0.05^Ba^	3.52 ± 0.16^Aa^	3.65 ± 0.34^Ba^	3.37 ± 0.21^Aa^
M:MI	0.19 ± 0.05^Aa^	0.16 ± 0.02^Aa^	0.16 ± 0.02^Aa^	0.12 ± 0.008^Aa^
M:MIII	0.68 ± 0.21^Bb^	1.06 ± 0.17^Aa^	0.99 ± 0.07^Ba^	0.56 ± 0.22^Ab^

*Parameters*: *AI* Amide I band, *CI* Crystallinity Index, *M:MI* Amide I + II/Hydroxyapatite (HA), *M:MIII* Amide III + Collagen/HA. In the rows, different capital letters indicated significant differences for radiation factor and different lower case letters indicated significant difference for experimental time interval. (*p* < 0.05)

The mean and standard deviation values for the mechanical test are shown in Table 2. There were decreased flexural modulus values in the irradiated groups compared with the respective control femurs (IR30 vs C30 and IR60 vs C60); however, for all groups evaluated, the time did not influence this property. No difference in flexural strength was identified between control and irradiated groups, and the time factor increased the flexural resistance results for both groups (C30 vs. C60 and IR30 vs. IR60). There was decreased elastic modulus in the irradiated groups compared with their respective controls (C30 vs. IR30 and C60 vs. IR60) and the time factor increased this property in the irradiated groups (IR30 vs. IR60). Vickers hardness decreased in the irradiated groups compared with control groups (IR30 vs. C30 and IR60 vs. C60), and the time factor increased the Vickers hardness in the control group (C30 vs. C60).

**Table 2:** The means and standard deviation values of biomechanics analysis in the femurs

Parameters	C30	IR30	C60	IR60
Flexural modulus (GPa)	53.7 ± 14.8 ^Aa^	65.5 ± 15.6 ^Ba^	86.6 ± 34.6 ^Aa^	95.0 ± 25.8 ^Ba^
Flexural strength (MPa)	12.9 ± 0.3 ^Ab^	9.8 ± 1.2 ^Ab^	12.5 ± 1.0 ^Aa^	11.0 ± 1.2 ^Aa^
Elastic modulus (GPa)	14.3 ± 2.8 ^Aa^	8.2 ± 2.7 ^Ba^	15.8 ± 3.1 ^Aa^	10.9 ± 4.0 ^Bb^
Vickers hardness (MPa)	84. ± 35.1 ^Ab^	58.3 ± 72.3 ^Ba^	147.6 ± 84.5 ^Aa^	52.0 ± 23.5 ^Ba^

In the rows, different capital letters indicated significant differences for radiation factor and different lower case letters indicated significant difference for experimental time interval. (*p* < 0.05)

## Discussion

Our study showed that ionizing radiation induced specific changes in the bone matrices, cortical microarchitecture, and collagen phase with changes in the mature/immature crosslinks ratio, and collagen/HA ratio. The evaluation over time showed some differences in the time intervals analyzed, suggesting that the increase in time after IR also dictated some features of bone quality. Radiotherapy is frequently used for curative or adjuvant cancer treatment, however, the intimate relations between the bone and soft-tissue tumors, or primary osseous lesions, may cause many complications in bone tissue. These outside effects on bone tissue seemed to be dependent on some factors such as dose and time of analyses [18].

The 30 Gy used in the present study was based in previous studies, which showed that a single high dose of IR led to bone damage, which allowed the effect of irradiation on bone to be evaluated [3, 21]. The fractionated irradiation protocol, recommended for use in human radiotherapy, is complex to perform in animal models. This is because the procedure requires multiple irradiations and repeated anesthesia, which is undesirable, as it compromises reproduction of the study, and increases the animal mortality rate [4]**.** In addition, the 30 and 60 days period after radiation was used as the endpoint of choice, because rodents are known to have a metabolic rate four to six times higher than that of humans [22, 23]. The post-radiation interval would thus be comparable to a follow-up period of 24–48 weeks (approximately six months) in a patient situation [22, 23], equal to the common latent period of late radiation complications [5, 6].

In the present study, histomorphometric analyses showed lower bone matrix levels in C30 compared with IR30. Some studies have reported that irradiation changed the bone turnover, significantly reduced vessel diameter [18] and could decrease remodeling process, which showed more bone matrix formation [24]. Thus, the higher matrix values shown in IR30 probably reflected changes and delay in the bone remodeling process. Moreover, IR60 did not show any increase in bone matrix compared with IR30, as shown in the control groups (C30 vs C60), supporting the hypothesis that IR would negatively compromise bone metabolism.

Lacunarity analyses showed that IR30 was more heterogeneous compared with C30 as regards the bone channel networks. This methodology is a general technique that can be applied to binary or quantitative data of any dimensionality, and it allows the determination of scale-dependent changes in spatial structure [18]. It also reveals the presence and range of self-similarity and can thus be considered a scale-dependent measure of heterogeneity and complexity [25]. Indeed, the significant difference in bone matrix area and lacunarity found represented alterations in bone microstructure and morphological characteristics in the irradiated group. This fact was in agreement with the findings of other studies that suggested the presence of severely disorganized bone matrix components [7, 8] including Haversian systems [18] after radiation.

In collagen maturity analysis, our results showed that there was a decreased enzymatic crosslink peak ratio in IR30 compared with C30. Studies have shown that ratio of these two bands corresponds to the number of enzymatic collagen cross-links present; specifically the non-reducible mature Pyr cross-links (interfibrillar) and the reducible immature DHNLN cross-links (intrafibrillar) found in bone [16]. The decreased ratio in IR30 suggested changes in the cross-link profile with increase in immature cross-links in relation to the mature cross-links [26]. This could disrupt the mature cross-links integrity, such as covalent hydroxypyridinium, leading to premature mechanical failure of the bone [26]. Furthermore, some molecular studies have reported radiation-induced changes within the mineral and organic the bone components [15, 27]. Evidence has shown that irradiation alters the degree of cross-linking within the collagen [28]. This occurs as a result of side chain decarboxylation of the collagen molecule, thus modifying the interaction or binding between the organic matrix and the HA mineral [29, 30].

Indeed, the crystallinity index and CT analysis showed that in IR60 had decreased mineral crystal compared with C60. This result suggested that IR increased the presence of large HA crystals and decreased the surface area in collagen fibrils [31] at the late time. It is also possible that the cell damage caused by radiation resulted in impaired and abnormal mineralization. This could be due to slowing down of the process, so that the resulting mineral would have time to develop larger, less carbonated crystallites than normal and abnormally crystalline mineral content [27]. M:MI showed no statistical difference between the groups. This suggested that IR reduced collagen maturity and crystallinity in the same proportions, thus without change in the ratio between the organic and inorganic matrix. Some studies in humans and animals have shown that IR impaired bone metabolism, leading to decreased bone mass [27, 32].

However, M:MIII showed that the matrix/mineral ratio increased in the Control groups and decreased in Irradiated groups when values at 30 days were compared with those at 60 days. The decreased mean values suggested that mineral composition had increased and/or the matrix content had decreased in Irradiated groups. Some studies have shown that in bone IR led to significant decrease in selected amino acids [33] and alterations in bone mineral composition, in which the irradiated tissue became hypermineralized, with an abnormally crystalline mineral [27]; this could be the explanation for our results.

The texture of apatite crystals, such as their size, shape, and collagen arrangement, are important in the establishment of the biomechanical and structural properties of bone [34]. Thus, the matrix alterations shown by the ATR-FTIR analysis, could explain our biomechanical results. In the present study, the IR groups showed lower values for flexural strength, elastic modulus and changes in bone stiffness, leading to greater susceptibility to fractures. The primary aspect of the irradiation-induced loss of fracture resistance could be due to complete loss of plastic deformation (intrinsic toughness) after irradiation [7]. In addition, the increased plastic (residual) strain during the post-yield deformation was mostly related to the collagen phase [8], while the mineral phase had minimal influence on plastic deformation in bone [23].

The Irradiated groups in the present study showed lower Vickers hardness values concomitant with less capability of undergoing plastic deformation in both time intervals, with values being almost 3 times lower in the time interval of 60 days. Studies have shown that irradiation induced bone embrittlement due the suppression of plasticity from fibrillar sliding and the consequent major losses in hardness [26]. This resulted from an increase in specific collagen cross-linking that raises the amount of bonds, and further exposures to irradiation could also cause molecular damage [35]. Moreover, irradiation exposure leads to the release of free radicals via radiolysis of water molecules in bone, which can severely degrade the collagen molecules in addition to restricting the fibrillar sliding mechanisms [26, 36].

The biomechanical properties such as Flexural modulus, Flexural strength, Elastic modulus, Vickers hardness are parameters that are used to evaluate bone fragility and strength [37, 38]. Changes in these indicators of bone biomechanical wholeness in the Irradiation groups, suggested many alterations including crystallization, mineralization disorders, collagen deformation, and therefore damage in bone property [37, 38]. In our study, changes in the biomechanical parameters of irradiated groups indicated considerable compromise of bone quality due to radiation; in other words, they showed degeneration, damaged bone wholeness, and that the bone had decreased resistance to fragility fractures.

Taken together, our findings revealed some previously unrecognized skeletal alterations in irradiated femurs and tibiae, relative to bone matrix components and architecture, which were not generally observed in a multi-modal in vivo evaluation technique. After irradiation, the bone matrix showed higher heterogeneity of the channel sizes and distribution - meaning that the bone channel network was altered due to radiation. Bone radiodensity was altered only in the longest time interval evaluated. However, the mechanical behavior was affected in both shorter and longest time intervals, with lower stiffness and altered post-yield deformation values. In addition, the collagen to HA ratio and maturation were altered by radiation, and some specific changes were also related to the increase in time after the effects of IR.

## Conclusions

The authors concluded that IR damaged collagen and hydroxyapatite, decreased bone radiodensity and stiffness in biomechanical tests. Moreover, the results suggested that the deleterious effects of IR increased in the late time points.

## Abbreviations

AI: Amide I band
ATR-FTIR: Attenuated Total Reflectance Fourier Transform Infrared Spectroscopy
C30: Control 30 days
C60: Control 60 days
CI: Crystallinity Index
CT: Computed tomography
FM: Flexural modulus
FS: Flexural strength
GPa: Gigapascals
H&E: Hematoxylin and eosin
HA: Hydroxyapatite
IR: Ionizing radiation
M:MI: Matrix-to-mineral ratio: Amide I + II/Hydroxyapatite
M:MIII: Amide III + Collagen/HA
MPa: Megapascals
ROI: Regions of interest
RX30: Irradiated 30 days
RX60: Irradiated 60 days
